# Microstructural Changes of the Human Brain from Early to Mid-Adulthood

**DOI:** 10.3389/fnhum.2017.00393

**Published:** 2017-08-07

**Authors:** Lixia Tian, Lin Ma

**Affiliations:** School of Computer and Information Technology, Beijing Jiaotong University Beijing, China

**Keywords:** fractional anisotropy (FA), axial diffusivity (AD), radial diffusivity (RD), multivariate pattern analysis, aging

## Abstract

Despite numerous studies on the microstructural changes of the human brain throughout life, we have indeed little direct knowledge about the changes from early to mid-adulthood. The aim of this study was to investigate the microstructural changes of the human brain from early to mid-adulthood. We performed two sets of analyses based on the diffusion tensor imaging (DTI) data of 111 adults aged 18–55 years. Specifically, we first correlated age with skeletonized fractional anisotropy (FA), mean diffusivity (MD), axial diffusivity (AD) and radial diffusivity (RD) at global and regional level, and then estimated individuals’ ages based on each DTI metric using elastic net, a kind of multivariate pattern analysis (MVPA) method that aims at selecting the model that achieves the best trade-off between goodness of fit and model complexity. We observed statistically significant negative age-vs-FA correlations and relatively less changes of MD. The negative age-vs-FA correlations were associated with negative age-vs-AD and positive age-vs-RD correlations. Regional negative age-vs-FA correlations were observed in the bilateral genu of the corpus callosum (CCg), the corticospinal tract (CST), the fornix and several other tracts, and these negative correlations may indicate the earlier changes of the fibers with aging. In brain age estimation, the chronological-vs-estimated-age correlations based on FA, MD, AD and RD were *R* = 0.62, 0.44, 0.63 and 0.69 (*P* = 0.002, 0.008, 0.002 and 0.002 based on 500 permutations), respectively, and these results indicate that even the microstructural changes from early to mid-adulthood *alone* are sufficiently specific to decode individuals’ ages. Overall, the current results not only demonstrated statistically significant FA decreases from early to mid-adulthood and clarified the driving factors of the FA decreases (RD increases and AD decreases, in contrast to increases of both measures in late-adulthood), but highlighted the necessity of considering age effects in related studies.

## Introduction

With the advent of diffusion tensor imaging (DTI), novel opportunities emerged for *in vivo* characterization of the brain’s white matter microstructure. To date, numerous studies have been performed on the maturation and aging of the human brain using such DTI metrics as fractional anisotropy (FA), mean diffusivity (MD), axial diffusivity (AD) and radial diffusivity (RD; Salat et al., 2005; Madden et al., 2009; Dennis and Thompson, 2013; Kulikova et al., 2015). According to these studies, FA increases and MD decreases with maturation (Qiu et al., 2008; Giorgio et al., 2010; Lebel et al., 2012; Krogsrud et al., 2016), and FA decreases and MD increases with aging in a majority of white matter tracts (Sullivan and Pfefferbaum, 2006; Charlton et al., 2008; Lebel et al., 2012; de Groot et al., 2016). In addition, FA changes are driven by the changes of AD and RD. Specifically, AD and RD decrease with maturation and increase with aging, and it is the greater relative changes of RD as compared to AD on both sides of the minimum that drive the FA changes (Giorgio et al., 2010; Hsu et al., 2010; Lebel et al., 2012; Sala et al., 2012; Kulikova et al., 2015).

Despite the former findings, the patterns and extent of the microstructural changes of the human brain from early to mid-adulthood remain largely unexplored. Though there have been many studies on the changes throughout life (Hasan et al., 2009; Westlye et al., 2010; Lebel et al., 2012; Sexton et al., 2014), changes with maturation and aging (at late adulthood) often played the predominant roles (e.g., driving the evolution curve) in those studies. For instance, dramatic changes with maturation and slow changes with aging of a measure would produce a relatively “early peak” of the measure, while slow changes with maturation and dramatic changes with aging of a measure would produce a relatively “late peak” of it. In other words, the changes of the human brain from early to mid-adulthood may largely be overwhelmed by those with maturation and aging, as the former may be relatively small as compared to the latter.

Investigating the microstructural changes of the human brain from early to middle adulthood will not only add to our knowledge about the changes throughout the lifespan, but also provide preliminary ideas about possible age effects in related studies. In fact, the effects of microstructural changes from early to mid-adulthood, if there are any, have not been considered carefully in related studies. For instance, in some studies including adult subjects covering large age span (e.g., of 19–59 years in the study by Welcome and Joanisse (2014) and of 25–58 years in the study by Boltzmann et al. (2017), possible age effects were not specially considered.

The aim of this study was to investigate the patterns and extent of the microstrucutural changes of the human brain from early to mid-adulthood. In addition to traditional statistical analyses, we further performed multivariate pattern analysis (MVPA) to investigate whether the microstructural changes of the human brain from early to mid-adulthood *alone* are sufficiently specific to decode individuals’ ages. Two recent studies demonstrated that individuals’ brain ages could be estimated at relatively high accuracy based on the DTI metrics by using MVPA methods (Mwangi et al., 2013; Erus et al., 2015). As child and adolescent subjects (whose brain structures undergo great changes) were included in both studies, relatively high estimating accuracies are expected. As compared to the changes in children and adolescents, the microstructural changes from early to mid-adulthood may be relatively small, and it is still unknown whether the microstructural changes from early to mid-adulthood *alone* are sufficiently specific to decode individuals’ ages indeed.

In this study, elastic net (E-Net) was used for brain age estimations. E-Net is a kind of MVPA method that aims at selecting the model that achieves the best trade-off between goodness of fit and model complexity by minimizing the residual sum of squares of estimating errors plus the penalty term. By imposing a sparsity requirement directly on the data space (rather than on kernel space, as is the fact for support vector machine and relevant vector machine), E-Net obtains predictive models that are easy to interpret without the need of feature selection beforehand. E-Net has been successfully applied to the predictions of individuals’ brain ages (Khundrakpam et al., 2015; Tian et al., 2016), behavior (Grosenick et al., 2013), and disorder status (Wan et al., 2011; Chiang et al., 2015).

The study was performed on the DTI data of 111 adults aged 18–55 years selected from a publicly released dataset. A cut off age of 55 years was chosen for the consideration that it was neither too low to exclude too many subjects and decrease the statistical power, nor too high to include old adults in Wolkorte et al. (2014) and Kodiweera et al. (2016). To test whether there were general changes in any of the four DTI metrics, we first correlated age with the mean of each DTI metric across the voxels within the FA skeleton mask obtained using standard tract-based spatial statistics (TBSS). Age was then correlated with the skeletonized maps of each DTI metric in a voxel-wise manner to test for regional microstructural changes. To test whether the microstructural changes from early to mid-adulthood *alone* are specific enough to decode individuals’ ages, we finally estimated individuals’ ages using elastic net based on each of the DTI metrics.

## Materials and Methods

### Dataset

The data used in the present study were selected from the publicly released dataset “the Nathan Kline Institute/Rockland Sample (NKI–RS)”^1^ (Nooner et al., 2012). The data acquisition was approved by the institutional review board of the Nathan Kline Institute. The initial release of the NKI-RS dataset included 207 participants, each of whom underwent multimodal brain scans and a battery of clinical assessments. One hundred and eleven healthy adult subjects aged 18~55 years (mean age: 35.00 ± 11.19 years, 71 males), whose DTI data were available, were included in the present study.

The MRI data were acquired using a 3.0 T SIEMENS Trio scanner. The DTI images were acquired using the following parameters: TR/TE = 10,000/91 ms, FOV = 256 mm, spatial resolution = 2.0 × 2.0 × 2.0 mm, slices = 58, 64 diffusion encoding directions, *b*-value = 1000 s/mm^2^ and 12 non-diffusion volumes. Further details about the image acquisition protocol could be found on the INDI website provided above. Other images not used in the present study will not be described here.

### Image Preprocessing and TBSS

FMRIB’s Diffusion Toolbox (FDT) in FMRIB’S Software Library (FSL)^2^ was used for image preprocessing. The images were first skull stripped and then were corrected for eddy currents. A diffusion tensor model was fit to each voxel to generate FA, MD, AD and RD images. The standard TBSS procedure (Smith et al., 2006) was applied in this study to obtain the skeletonized images of the four DTI metrics. Specifically, all subjects’ FA images were first nonlinearly aligned to the FA template in the MNI space^3^. The aligned FA images were averaged to create a mean FA image, which was then thinned to create a skeletonized mean FA image, and a skeleton mask was finally created by thresholding the mean FA skeleton with FA ≥ 0.3 (Smith et al., 2006; Chiang et al., 2009; Mwangi et al., 2013). Here, we used a threshold of FA ≥ 0.3 mainly for consistency with the study by Mwangi et al. (2013), which was also based on the dataset “NKI–RS”. In addition, false positives may be reduced with the use of a higher FA threshold, as voxels which are primarily gray matter or cerebral spinal fluid in some subjects would be largely excluded (Smith et al., 2006). Each subject’s (aligned) FA image was finally projected onto the skeleton by filling the skeleton with FA values from the nearest relevant tract center. The skeletons for the MD, RD and AD images were created by first nonlinearly aligning to the FA template in the MNI space and then projecting onto the mean FA skeleton. All further analyses were based on the skeletonized images of the DTI metrics.

### DTI Metric-vs-Age Correlation

We first performed global-level statistical analyses to test whether there were general changes in any metric. We obtained the mean of each DTI metric (across the voxels within the FA skeleton mask) of each subject using “fslmeants” command in FSL, and then each mean DTI metric was correlated with age across subjects. As the DTI metrics were reported to change nonlinearly throughout life (Hasan et al., 2009; Westlye et al., 2010; Lebel et al., 2012), we also modeled the mean of each DTI metric as a quadratic function of age (*A* × (*age − B*)^2^ + *C*) to check whether age-related changes of the DTI metrics from early to mid-adulthood can be well fitted using a nonlinear model.

To test for regional microstructural changes, age was then correlated with each DTI metric of the voxels within the FA skeleton mask in a voxel-wise manner. For this purpose, 5000 permutations were performed on the skeletonized images of each DTI metric across subjects using “randomize” command in FSL. The voxel-wise correlation maps were thresholded at *p* < 0.05 (family-wise error (FWE) corrected) and cluster size ≥ 10 mm^3^. Statistically significant clusters were filled using the “TBSS_fill” command in FSL beforehand for display convenience.

### Brain Age Estimation

We then estimated individuals’ ages based on each DTI metric to test whether the microstructural changes of the human brain are specific enough to decode individuals’ ages using E-Net. Specifically, for each DTI metric, we entered the metric of all voxels within the FA skeleton mask into the following linear model in a voxel-wise manner:

(1) $$y\;=\;\sum_{j\;=\;1}^{d}\beta_{j}x_{j}+\beta_{0}+\varepsilon$$

where *y* is the age of the subject; *d* is the number of variables, and here *d* was 97602; *x*_j_s (*j* = 0, 2, …, *d*) are voxel-wise DTI metrics; *β*_j_s (*j* = 0, 2, …, *d*) are the model parameters, and *ε* is the error term. Penalized least squares approach with E-Net penalty (a weighted sum of the least absolute shrinkage and selection operator (LASSO) penalty and ridge penalty) was used to estimate the parameters of the model (Zou and Hastie, 2005). The aim of the approach is to minimize the E-Net cost function:

(2) $$\sum_{i\;=\;1}^{N}{\left(y_{i}-\sum_{j\;=\;1}^{d}\beta_{j}x_{ij}\;-\;\beta_{0}\right)}^{2}+\lambda \sum_{j\;=\;1}^{d}\left(\alpha |\beta_{j}|+\;0.5(1\;-\;\alpha ){\left(\beta_{j}\right)}^{2}\right)$$

where *y*_i_ is the age of subject *i*; *N* is the number of subjects; *x*_ij_ is the *j*th variable (DTI metric of a voxel) of subject *i*; λ and *α* are regularization parameters. The LASSO penalty $\sum_{j\;=\;1}^{d}\left|\beta_{j}\right|$ leads to sparse models by setting some parameters (*β*_j_s) to zero, and the ridge penalty $\sum_{j\;=\;1}^{d}{\left(\beta_{j}\right)}^{2}$ encourages the *β*_j_s for the correlated variables (voxel-wise DTI metrics) to have approximately the same value. The regularization parameters λ and *α* set the sparsity of the model and tune the ratio between the ridge and LASSO penalties, respectively.

In this study, E-Net was performed using Glmnet (Friedman et al., 2010)^4^. Leave one out cross validation (LOOCV) was used to estimate the performance of the predictors. Within each of the 111 LOOCV loops, one sample was set as the test sample, all the other 110 samples were set as the training samples, and the brain age of the test sample was estimated using the predictive model trained completely on the 110 training samples. With the use of LOOCV, the test sample was assumed to be independent of the training samples. It should be noted that this assumption of independence may sometimes not strictly hold, though LOOCV has been widely accepted in the region (Dosenbach et al., 2010; Mwangi et al., 2013; Tian et al., 2016). A more reliable way may be training the model using one dataset and evaluating the model using another dataset, if a second dataset were available.

The regularization parameters λ and α were optimized for each LOOCV loop. Specifically, for each training-set (including 110 samples), we tried 10 α values (α ∈ [0.5, 0.95] in steps of 0.05, we set α ≥ 0.5 to give more weight for the LASSO penalty) and 100 λ values. Based on each λ-α pair, “inner” cross validation was then performed, and the λ-α pair that maximize the correlation between the estimated and chronological ages (based on the 110 training samples) were selected as the optimal regularization parameters for the current training samples (Tian et al., 2016). Brain age estimation was then performed on the corresponding testing data using the optimal λ-α pair.

Pearson’s correlation coefficient and mean absolute error (MAE) between the estimated and chronological ages were used to evaluate the performance of the estimations. Using 500 permutations, we estimated how likely we were to observe the same brain age estimation performance by chance. Specifically, the chronological ages of the subjects were randomly permuted 500 times (randomly assign the age of one subject to another), and the entire estimation process was carried out with each set of the permuted ages.

## Results

**Figure 1:**
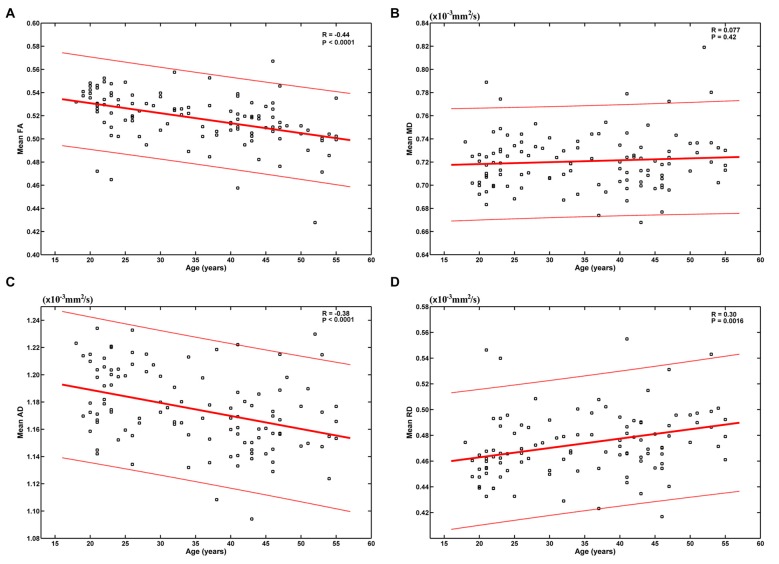
Relationship between age and the mean of each diffusion tensor imaging (DTI) metric. Age was significantly negatively correlated with mean fractional anisotropy (FA) **(A)** and axial diffusivity (AD) **(C)**, and positively correlated with mean radial diffusivity (RD) **(D)**. No statistically significant correlation was found between age and mean mean diffusivity (MD) **(B)**.

The results based on the global-level statistical analyses are shown in Figure 1. It can be seen that age was significantly negatively correlated with mean FA (*R* = −0.44, *P* < 0.0001) and AD (*R* = −0.38, *P* < 0.0001), and positively correlated with mean RD (*R* = 0.30, *P* = 0.0016). These results indicate an overall decrease of FA and AD and increase of RD from early to mid-adulthood. No statistically significant correlation was found between age and mean MD (*R* = 0.077, *P* = 0.42).

When we modeled the mean of each DTI metric as a quadratic function of age (*A* × (*age − B*)^2^ + *C*), the fitting parameters indicate inverse U-shaped age-related FA changes and U-shaped age-related MD, AD and RD changes (Supplementary Figure S1). However, FA was estimated to peak at −18.92 years (Supplementary Figure S1A), which was unreasonable with no doubt. RD was estimated to reach its minimum at 18.87 years (Supplementary Figure S1D), and this bottom was also much earlier than those reported in literatures (Lebel et al., 2010, 2012).

**Figure 2:**
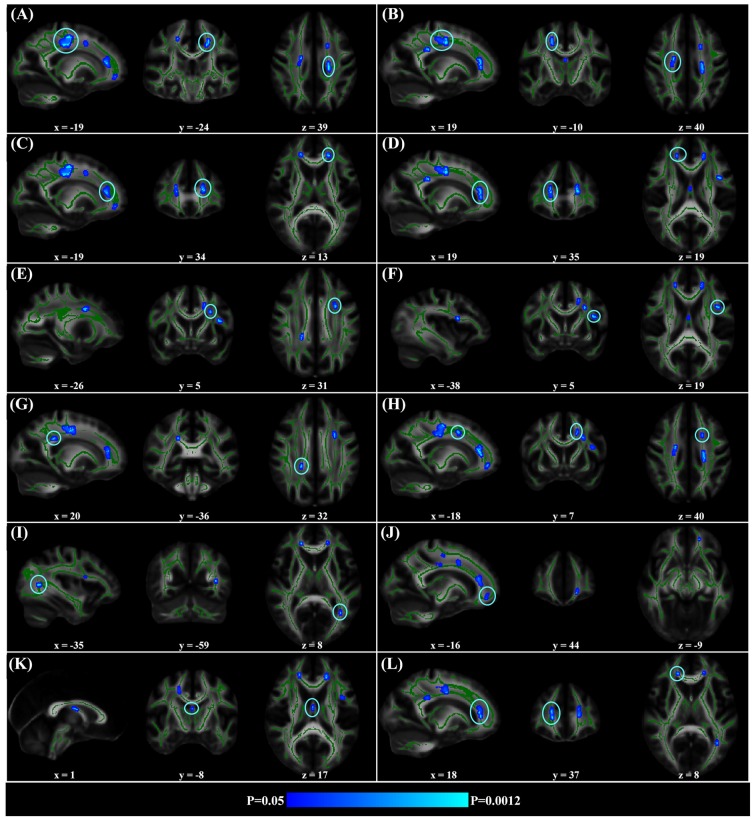
Statistically significant negative age-vs-fractional anisotropy (FA) correlations across subjects. The threshold was *p* < 0.05 (FWE corrected) and cluster size ≥10 mm^3^. The correlation map was superimposed on the FA template in the MNI space (http://fsl.fmrib.ox.ac.uk/fsl/fslwiki/FMRIB58_FA). Green color indicates the FA skeleton mask, and blue-light-blue color indicates statistically significant negative correlations. Statistically significant negative age-vs-FA correlations were observed in the bilateral corticospinal tract (CST) **(A,B)**, the genu of the corpus callosum (CCg) **(C,D,J,L)**, the fornix **(K)**, the left superior longitudinal fasciculus (SLF) **(E,F,H)**, the left inferior longitudinal fasciculus (ILF) **(I)**, and the right splenium of the CC **(G)**, as were highlighted by circles when there were more than one cluster on the slice. No statistically significant positive age-vs-FA correlation was observed.

**Figure 3:**
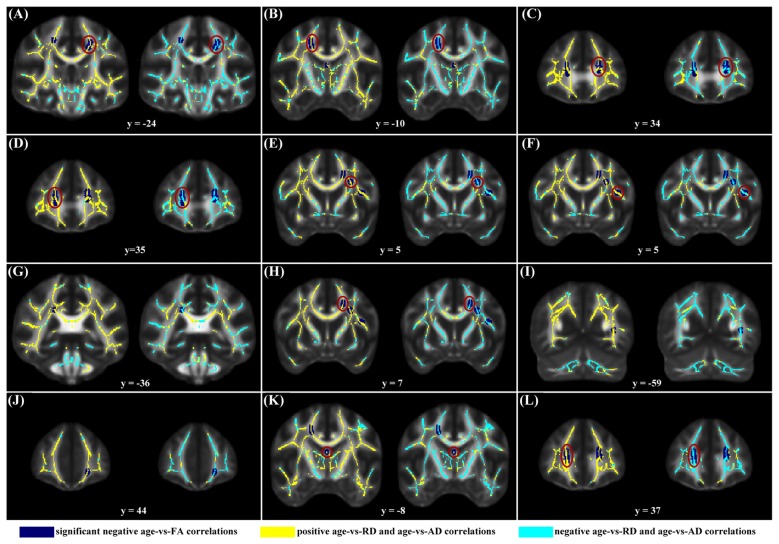
Maps of age-vs-radial diffusivity (RD) and age-vs-axial diffusivity (AD) correlations across subjects in clusters exhibiting statistically significant age-vs-FA correlations. The clusters include: the bilateral CST **(A,B)**, the genu of the CC **(C,D,J,L)**, the fornix **(K)**, the left SLF **(E,F,H)**, the left ILF **(I)**, and the right splenium of the CC **(G)**. Age-vs-RD and age-vs-AD correlations were shown on the left and right sides, respectively, of each subfigure. The dark blue color indicates statistically significant negative age-vs-FA correlations; the yellow color indicates positive age-vs-RD and -AD correlations; the light blue color indicates negative age-vs-RD and -AD correlations. To illustrate the pattern (positive or negative), rather than the strength, of age-vs-RD and -AD correlations, the two correlation maps were not thresholded. It can be observed that nearly all statistically significant negative age-vs-FA correlations were due to positive age-vs-RD and negative age-vs-AD correlations, and the only exception was the age-vs-FA correlation in the fornix **(K)**, where the age-vs-AD correlation was also positive.

When correlating age with FA in a voxel-wise manner, statistically significant negative correlations (*P* < 0.05, FWE corrected, cluster size ≥10 mm^3^) were observed in such fibers as the bilateral corticospinal tract (CST), the genu of the corpus callosum (CCg), the fornix, the left superior longitudinal fasciculus (SLF) and inferior longitudinal fasciculus (ILF), and the right splenium of the corpus callosum (CCs), and no statistically significant positive age-vs-FA correlation was observed (Figure 2, Table 1). Nearly all of the negative age-vs-FA correlations were associated with negative age-vs-AD and positive age-vs-RD correlations (Figure 3), and the only exception was the correlation in the fornix, which was associated with positive correlations with both RD and AD (Figure 3K). Statistically significant age-related correlations with MD, AD and RD were found in the corpus callosum (CC) and the CST (Supplementary Figures S2, S3 and S4, Table 2).

**Table 1:** Statistically significant age-vs-fractional anisotropy (FA) correlations.

Region	Cluster (mm^3^)	Coordinate (peak)	R (peak)	Region	Cluster (mm^3^)	Coordinate (peak)	R (peak)
**L. CST**	149	(−19, −24, 39)	−0.56	**R.CCs**	19	(20, −36, 32)	−0.52
**R. CST**	82	(19, −10, 40)	−0.55	**L. SLF**	18	(−18, 7, 40)	−0.51
**L. CCg**	76	(−19, 34, 13)	−0.54	**L. ILF**	15	(−35, −59, 8)	−0.53
**R. CCg**	35	(19, 35, 19)	−0.49	**L. CCg**	15	(−16, 44, −9)	−0.50
**L. SLF**	28	(−26, 5, 31)	−0.51	**fornix**	13	(1, −8, 17)	−0.50
**L. SLF**	22	(−38, 5, 19)	−0.56	**R. CCg**	13	(18, 37, 8)	−0.48

*L, left; R, right; CST, corticospinal tract; CCg, genu of corpus callosum; SLF, superior longitudinal fasciculus; ILF, inferior longitudinal fasciculus; CCs, splenium of corpus callosum. The threshold was P < 0.05 (FWE corrected), and cluster size ≥10 mm^3^*.

**Table 2:** Statistically significant age-vs-mean diffusivity (MD), -axial diffusivity (AD) and -radial diffusivity (RD) correlations.

Region	Cluster (mm^3^)	Coordinate (peak)	R (peak)	Region	Cluster (mm^3^)	Coordinate (peak)	R (peak)
**Correlations with MD**
**L. CST**	15	(−6, −21, −14)	−0.62	**R. CST**	10	(18, −9, 40)	0.52
**L. CCg**	11	(−7, 23, −5)	0.62				
**Correlations with AD**
**L. CST**	16	(−12, −25, −12)	−0.59	**L. CST**	11	(−24, −19, 0)	−0.58
**L. CST**	14	(−7, −22, −14)	−0.64				
**Correlations with RD**
**R. CST**	39	(19, −11, 39)	0.56	**L. CCb**	19	(−15, 9, 32)	0.49
**L. CCg**	27	(−18, 32, 17)	0.52	**R. CCg**	18	(7, 24, −5)	0.64
**R. CST**	23	(21, −16, 43)	0.50	**L. CCg**	14	(−7, 23, −5)	0.63
**R. CCg**	21	(19, 37, 14)	0.50	**R. CST**	10	(19, −22, 37)	0.48

*L, left; R, right; CST, corticospinal tract; CCg, genu of the corpus callosum; CCb, body of the corpus callosum. The threshold was P < 0.05 (FWE corrected), and cluster size ≥10 mm^3^*.

**Figure 4:**
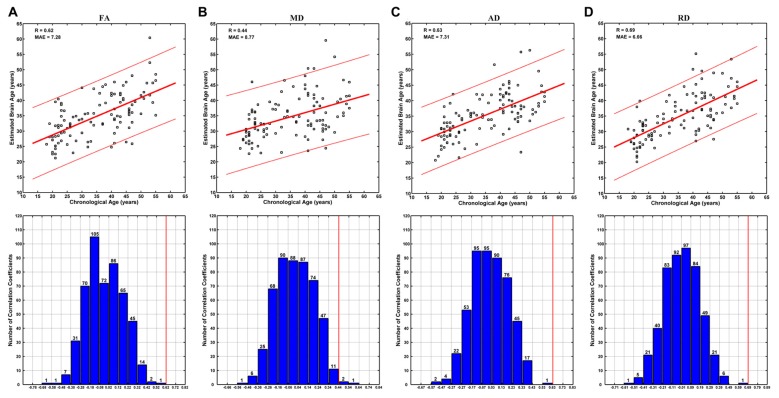
Brain age estimations based on FA** (A)**, mean diffusivity (MD) **(B)**, AD **(C)** and RD** (D).** The plot of estimated vs. chronological ages based on each DTI metric was shown on the upper panels, and predictions based on 500 permutations were shown on the lower panels of each subfigure. The predictions were evaluated by the Pearson’s correlation coefficients between the estimated and permuted chronological ages. The red lines on the lower panels indicate the estimation based on non-permuted ages (corresponding to the *R*-values on the upper panels). Each bar on the lower panels indicates the number of correlations (between the estimated and permuted chronological ages) within the given range, which was set relative to the correlation based on non-permuted ages (indicated by a red line) at a step of 0.1. It can be seen that no better prediction was obtained in any of the 500 permutations based on FA, AD and RD, and three better estimations were observed based on MD.

Brain ages could be estimated at relatively high accuracy based on each DTI metric. Specifically, no better brain age estimation was obtained in any of the 500 permutations based on FA, AD and RD, and three better estimations were observed among 500 permutations based on MD (Figure 4). The correlations between the estimated and chronological ages based on FA, MD, AD and RD were 0.62, 0.44, 0.63 and 0.68, respectively (MAE = 7.28, 8.77, 7.31 and 6.66 years; Figure 4).

## Discussion

In contrast to maturation and aging of the human brain, structural and functional changes of the human brain from early to mid-adulthood have rarely been specially studied. In this study, we investigated microstructural changes of the human brain from early to mid-adulthood. Based on the DTI data of 111 adults aged 18–55 years, we observed statistically significant negative age-vs-FA correlations, as well as statistically significant age-related correlations with other DTI metrics, from early to mid-adulthood. Further brain age estimations showed that the changes were specific enough to decode individuals’ ages.

### FA Decreases

In this study, we observed both global and regional negative age-vs-FA correlations (Figures 1A, 2, Table 1) and suggest that the correlations indicate microstructural aging of the human brain from early to mid-adulthood. FA decrease is an important characteristic of aging. Decreases in FA in the elderly have been suggested to signify worsening white matter integrity, and quite a few studies linked decreases in FA in the elderly to mild demyelination and loss of myelinated axons in them (Benedetti et al., 2006; Ardekani et al., 2007; Grieve et al., 2007; Charlton et al., 2008; Madden et al., 2009; Lebel et al., 2010; Ly et al., 2014). Accordingly, the statistically significant negative age-vs-FA correlations observed in the current study (Figures 1A, 2, Table 1) may indicate an overall microstructural aging of the human brain from early to mid-adulthood.

Regional negative age-vs-FA correlations were observed in such fibers as the CCg, the CST, the fornix, the SLF and the ILF (Figure 2). Each of the fibers has been reported to show statistically significant FA decreases in former aging studies (Abe et al., 2002; Zahr et al., 2009; Teipel et al., 2010; Lövdén et al., 2014). Specifically, the CCg, which connects the bilateral prefrontal lobes, have been reported to be rather susceptible to aging (Pfefferbaum et al., 2000; Abe et al., 2002; Bhagat and Beaulieu, 2004; Ota et al., 2006; Hsu et al., 2010; Zhang et al., 2010). The early aging of CCg has been related to the fact that up to 20%–30% of its axons are unmyelinated (Lamantia and Rakic, 1990; Pagani et al., 2008). FA declines in the CCg in the elderly have been reported to be associated with lower perceptual speed and longer episodic retrieval reaction time (Schulte et al., 2005; Bucur et al., 2008), as well as poor performance in reasoning in them (Monge et al., 2016). CST is a major neural tract in the human brain for motor function. Numerous studies on aging reported FA decreases in the CST (Terao et al., 1994; Lövdén et al., 2014; Vik et al., 2015), and these decreases have been reported to be accompanied by reduction in the number of myelinated fibers and thinning of the tissue it originates (Terao et al., 1994). FA decreases in the CST have been reported to contribute much to decreases of perceptual speed in the elderly (Lövdén et al., 2014; Johnson et al., 2015; Vik et al., 2015). The SLF connects the temporo-parietal junction area and parietal lobe with the frontal lobe (Makris et al., 2005), and its FA decreases in the elderly have been linked with poor performance in cognitive tasks in them (Perry et al., 2009; Stamatakis et al., 2011; Lockhart et al., 2012; Sasson et al., 2012). Quite a few studies on aging reported age-related FA decreases in the fornix (Pagani et al., 2008; Stadlbauer et al., 2008; Lee et al., 2009; Zahr et al., 2009; Sullivan et al., 2010), a part of the limbic system important for episodic memory recall (Tsivilis et al., 2008), and declines of memory performance in the elderly have been reported to be associated with FA decreases in the fornix (Li et al., 2016; Oishi and Lyketsos, 2016). Closely relevant to the current results, the fornix, the CCg, the CCs and the ILF have been reported to be the four fibers that exhibit the earliest FA decreases in the lifespan study by Lebel et al. (2012). According to these former findings, we suggest that the statistically significant negative age-vs-FA correlations in the CCg, the CST, the fornix, the SLF and the ILF observed in the current study may indicate that the fibers are the earliest to change with aging.

### Changes in Other DTI Metrics

In addition to FA decreases, aging of the human brain has also been associated with MD increases (Benedetti et al., 2006; Lebel et al., 2012; Ly et al., 2014). Age-vs-MD correlations in the current study, however, were not statistically significant at global level (Figure 1B) and relatively weaker than those with FA at regional level (Supplementary Figure S2, Table 2). We suggest that age-related MD increases might lag behind age-related FA decreases. This suggestion is supported by reports that MD minima significantly lags behind FA peaks, with minimum MD values occurring 6–18 years after FA peaks (Lebel et al., 2010, 2012).

The negative age-vs-FA correlations in the current study were associated primarily with negative age-vs-AD and positive age-vs-RD correlations (Figures 1A,C,D, 3). This finding is consistent with the reports of a study by Kodiweera et al. (2016), in which significant RD increases and AD decreases were observed in middle-aged adult brains. Both RD and AD have been reported to decrease with development, reach a minimum and then increase in later life (Lebel et al., 2010, 2012). RD increases and AD decreases in the elderly have often been linked to age-related demyelination (Song et al., 2002, 2005; Bennett et al., 2010) and axonal shrinkage (Song et al., 2003; Bennett et al., 2010), respectively. We speculate that the present finding of statistically significant negative age-vs-AD and positive age-vs-RD correlations may reflect an RD-preceding-AD sequence of change of the two metrics, with AD having not reached its respective minimum, while RD has already passed its minimum at the time. This speculation is supported by the report that RD reaches its minimum and begins to increase before AD (Lebel et al., 2010). If our suggestion of “RD-preceding-AD” hypothesis were valid, we suggest that our finding of the fornix being the only region exhibiting AD increase (Figure 3K) might be due to its earlier change with aging relative to other fibers: AD increases in the fornix begin at an earlier time, when other fibers still exhibit AD decreases. This suggestion is consistent with former studies that reported the earliest maturation and degeneration of the fornix (Lebel et al., 2012). Further studies based on longitudinal data, rather than cross-sectional data, as was used in the current study, are needed to test the hypothesis.

### Brain Age Estimation

Using multivariate pattern analyses, several studies have been performed on brain age estimations based on MRI and reported relatively high accuracies (Dosenbach et al., 2010; Brown et al., 2012; Mwangi et al., 2013; Erus et al., 2015; Tian et al., 2016). Of particular interest, the study by Mwangi et al. (2013) was based on DTI data of subjects aged 4–85 years, and the study by Erus et al. (2015) was based on DTI data of subjects aged 8–22 years. As both studies included child and adolescent subjects, whose brain structures undergo great changes, successful brain age estimations are expected.

The current study included only young and middle-aged adults, whose brain structures have traditionally been deemed to be relatively stable. Our results indicate that, in contrast to the opinion that the microstructural changes from early to mid-adulthood are rather subtle as to be negligible, these changes were specific enough to decode individuals’ ages (Figure 4). In combination with the findings of statistically significant age-vs-DTI-metrics correlations, the present results highlight the necessity of considering age effects in related studies. Specifically, in studies including adult subjects covering large age span (Welcome and Joanisse, 2014; Boltzmann et al., 2017), possible age effects should be considered carefully, otherwise spurious results possibly reflecting age-effects, rather than the effect under investigation, may be obtained.

The brain age estimation based on FA was not as good as that based on RD (Figure 4). Specifically, the correlation between the estimated and chronological ages based on FA (0.62) was weaker than that based on RD (0.68), and the MAE based on FA (7.28 years) was larger than that based on RD (6.66 years). This is a bit unexpected, as the age-vs-FA correlation was the strongest at the global-level (Figure 1), and the number of statistically significant voxels was the largest in voxel-wise statistical analyses (Tables 1, 2). We suggest that strong FA-vs-age correlations do not necessarily mean better brain age estimations. That is, if there existed strong associations among the FA changes of different voxels, for an extreme example, the FA changes of all statistically significant voxels were fully correlated with each other, then the contributions of all statistically significant voxels to brain age estimations would be just the same as that of a single voxel. This suggestion is in line with the finding that the changes of FA in a tract often go together with changes in other tracts, while the changes of MD are weakly associated among each other (Lövdén et al., 2014). In addition, the present results were consistent with those in the study by Mwangi et al. (2013), in which brain age estimation based on FA was also poorer than that based on RD. Further studies are needed to evaluate the hypothesis.

### Methodological Issues

Quite a few studies reported nonlinear changes of the DTI metrics in most white matter tracts of the human brain throughout life (Hasan et al., 2009; Westlye et al., 2010; Lebel et al., 2012; Sexton et al., 2014). In those studies, nonlinear models work well, as substantial microstructural changes of the human brain occur with maturation and aging, and the changes are obviously nonlinear throughout life. In those studies, it was often the changes with maturation and aging that play predominant roles (e.g., driving the evolution curve; Lebel et al., 2012; Zhao et al., 2015), though the maximum/minimum of the DTI metrics were often reported to occur during 25–45 years old (Lebel et al., 2010, 2012).

In the current study, we chose to use linear models because of the following two facts: (1) we observed no obvious nonlinear relationship between age and any DTI metric by visual inspection (Figure 1); and (2) when we modeled the mean of each DTI metric as a quadratic function of age (*A* × (*age − B*)^2^ + *C*), absurd results were obtained (Supplementary Figure S1): based on the current dataset, the maximum of mean FA was estimated to occur at −18.92 years (Supplementary Figure S1A), and the minimum of mean RD was estimated to occur at 18.87 years (Supplementary Figure S1D), which was much earlier than those reported in literatures (Lebel et al., 2010, 2012). These results based on nonlinear models indicate that wrong conclusions may be drawn if nonlinear models were used inappropriately. With the use of linear models, the nonlinear changes of the DTI metrics with age, if there are, could not be detected. Therefore, it should be strengthened that the present results reflect only “overall tendencies” of the changes of the DTI metrics (e.g., FA decreases with age) from early to mid-adulthood.

There are several other methodical issues that should be addressed. First, the current study was based on cross-sectional data, rather than longitudinal data. As cross-sectional data can provide only one measurement per subject, possible bias may occur. In fact, we could not rule out the possibility that the current results may, to some degree, be influenced by such factors as cohort effects, which are inevitable in studies based on cross-sectional data. Future longitudinal studies may be better able to depict the trajectories of the microstructural changes of the human brain from early to mid-adulthood. Second, the estimation errors of the DTI metrics in crossing fibers are not negligible. Therefore, the statistically significant regional age-related correlations observed in the current study, especially those in crossing fibers, should be further evaluated, for instance, by incorporating measures of intervoxel coherence (Bennett et al., 2010). Third, with the use of TBSS, later statistical analyses and brain age estimations were all restricted to major white matter tracts. TBSS may benefit reducing false positives (Smith et al., 2006; Mwangi et al., 2013), but some true positives may be excluded, as it is possible that age-related correlations exist in regions away from major white matter tracts. Finally, gender effects were not specially considered in the current study, as there was no significant difference in age between the two genders (*t* = 1.08, *p* = 0.28). Indeed, when we included gender as a covariate into our global-level statistical analyses, little change was observed on the age-vs-FA, -MD, -AD and -RD correlations: they changed from −0.44, 0.077, −0.38 and 0.30 to −0.43, 0.078, −0.36 and 0.29, respectively.

## Conclusions

We observed that the negative age-vs-FA correlations were associated primarily with negative age-vs-AD and positive age-vs-RD correlations. In addition, we observed earlier changes of such fibers as the CCg, the CST, the fornix and the SLF with aging relative to other fibers. The brain age estimation results showed that even the microstructural changes from early to mid-adulthood *alone* are sufficiently specific to decode individuals’ ages. Overall, this study provided convincing evidence that considerable microstructural changes of the human brain occur from early to mid-adulthood, and it is necessary to consider age effects in related studies.

## Author Contributions

LT and LM designed the study; LT and LM analyzed the data; LT and LM wrote the article.

## Conflict of Interest Statement

The authors declare that the research was conducted in the absence of any commercial or financial relationships that could be construed as a potential conflict of interest. The reviewer DM and handling Editor declared their shared affiliation, and the handling Editor states that the process nevertheless met the standards of a fair and objective review.

## Footnotes

^1^http://fcon_1000.projects.nitrc.org/indi/pro/nki.html

^2^http://www.fmrib.ox.ac.uk/fsl/

^3^www.fmrib.ox.ac.uk/fsl/data/FMRIB58_FA

^4^http://statweb.stanford.edu/~tibs/lasso.html

## Abbreviations

AD: axial diffusivity
CC: corpus callosum
CCg: genu of the corpus callosum
CCs: splenium of the corpus callosum (CCs)
CST: corticospinal tract
DTI: Diffusion tensor imaging
E-Net: elastic net
FA: fractional anisotropy
FSL: Fmrib’S Software Library
FWE: family-wise error
ILF: inferior longitudinal fasciculus
LASSO: least absolute shrinkage and selection operator
LOOCV: leave one out cross validation
MAE: mean absolute error
MD: mean diffusivity
RD: radial diffusivity
SLF: superior longitudinal fasciculus.
