# Supplementary material for: Microstructural Changes of the Human Brain from Early to Mid-Adulthood
Source: Front Hum Neurosci. 2017 Aug 7;11:393. doi: 10.3389/fnhum.2017.00393 (PMC5545923; doi:10.3389/fnhum.2017.00393)
Supplement: Supplementary file 1 [file Presentation_1.pdf]

Supplementary Materials

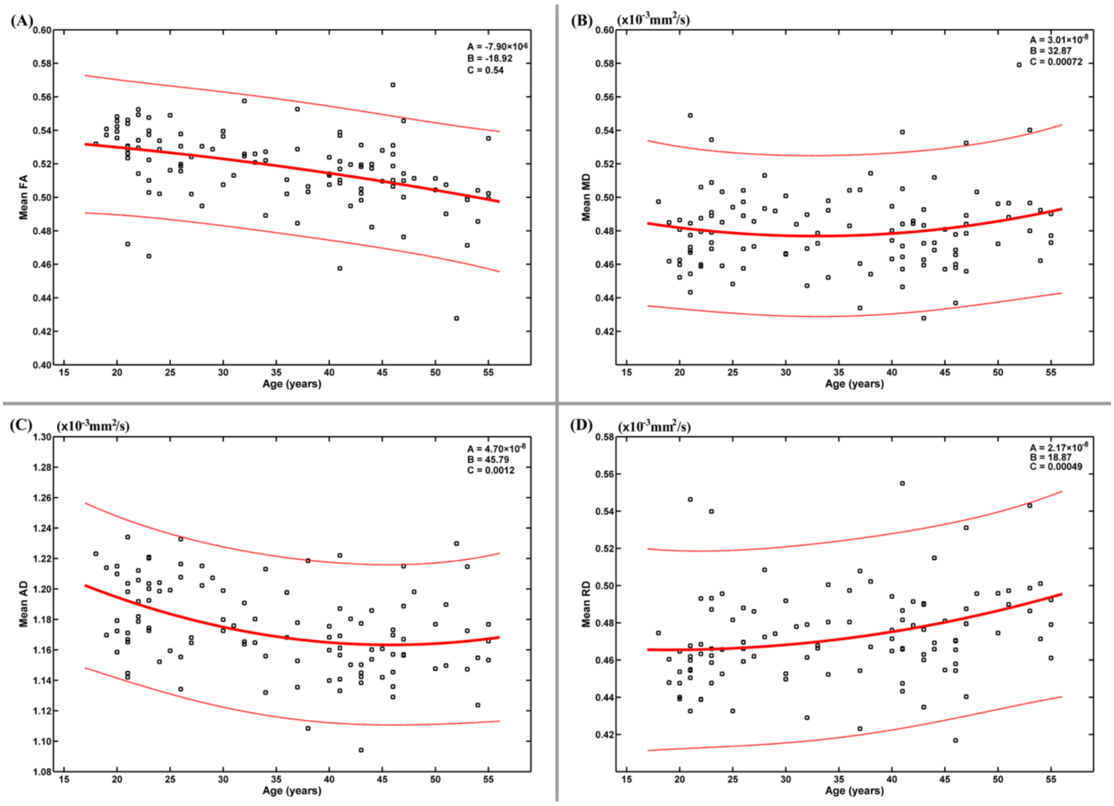

Fig. S1. Changes of mean FA (A), MD (B), AD (C) and RD (D) with age represented by quadratic curves. *The mean of each DTI metric was modeled as a quadratic function of age (* $A \times (age - B)^2 + C$ *). The fitting parameters indicate inverse U-shaped age-related FA changes and U-shaped age-related MD, AD and RD changes (Fig. S1). However, FA was estimated to peak at -18.92 years (Fig. S1(A)), which is unreasonable with no doubt. RD was estimated to reach its minimum at 18.87 years (Fig. S1(D)).*

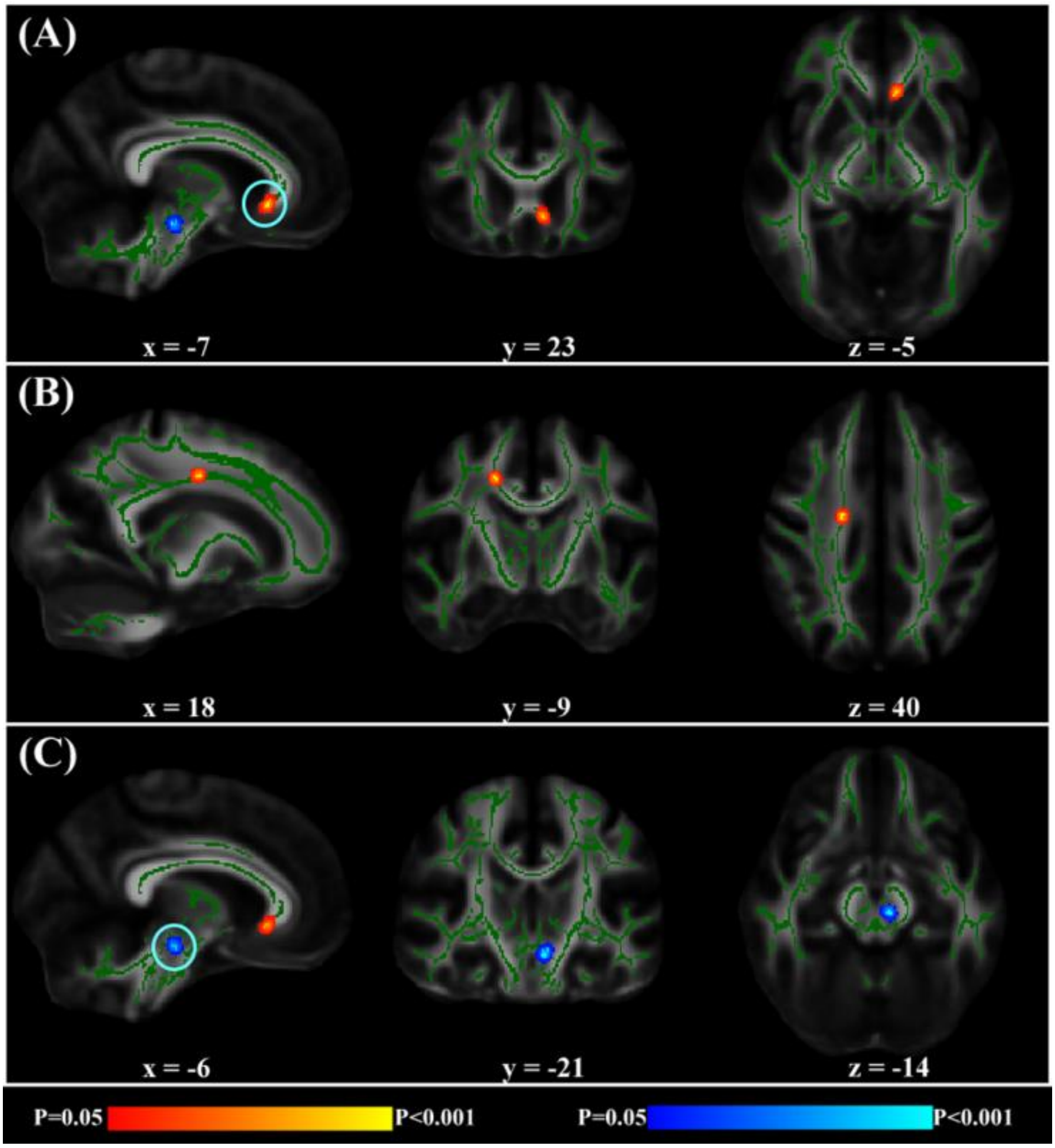

Fig. S2. Significant age-vs-MD correlations across subjects. *The threshold was $p < 0.05$ (FWE corrected), and only clusters not smaller than 10 mm^3 were shown. The correlation map was superimposed on the FA template in the MNI space (http://fsl.fmrib.ox.ac.uk/fsl/fslwiki/FMRIB58_FA). Green color indicates the FA skeleton mask, blue-light-blue color indicates significant negative correlations, and red-yellow color indicates significant positive correlations. Significant age-vs-MD correlations were observed in the left genu of the corpus callosum (A) and the bilateral corticospinal tract (B, C), as were highlighted by circles when there were more than one cluster on the slice.*

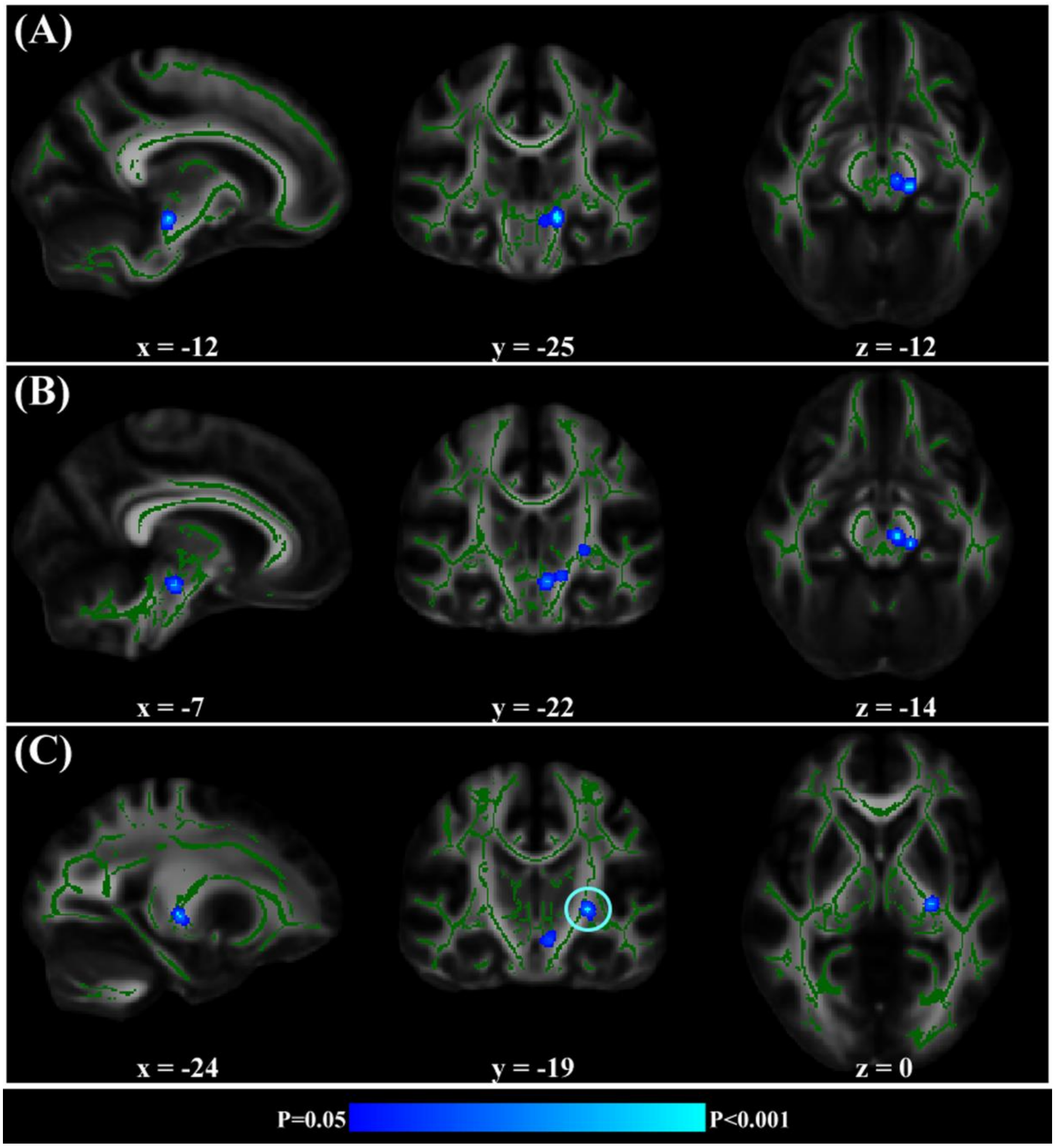

Fig. S3. Significant negative age-vs-AD correlations across subjects. *The threshold was $p < 0.05$ (FWE corrected), and only clusters not smaller than 10 mm^3 were shown. The correlation map was superimposed on the FA template in the MNI space (http://fsl.fmrib.ox.ac.uk/fsl/fslwiki/FMRIB58_FA). Green color indicates the FA skeleton mask, and blue-light-blue color indicates significant negative correlations. No significant positive correlation was observed. Significant negative age-vs-AD correlations were observed in the left corticospinal tract (A, B, C), as was highlighted by a circle when there were more than one cluster on the slice.*

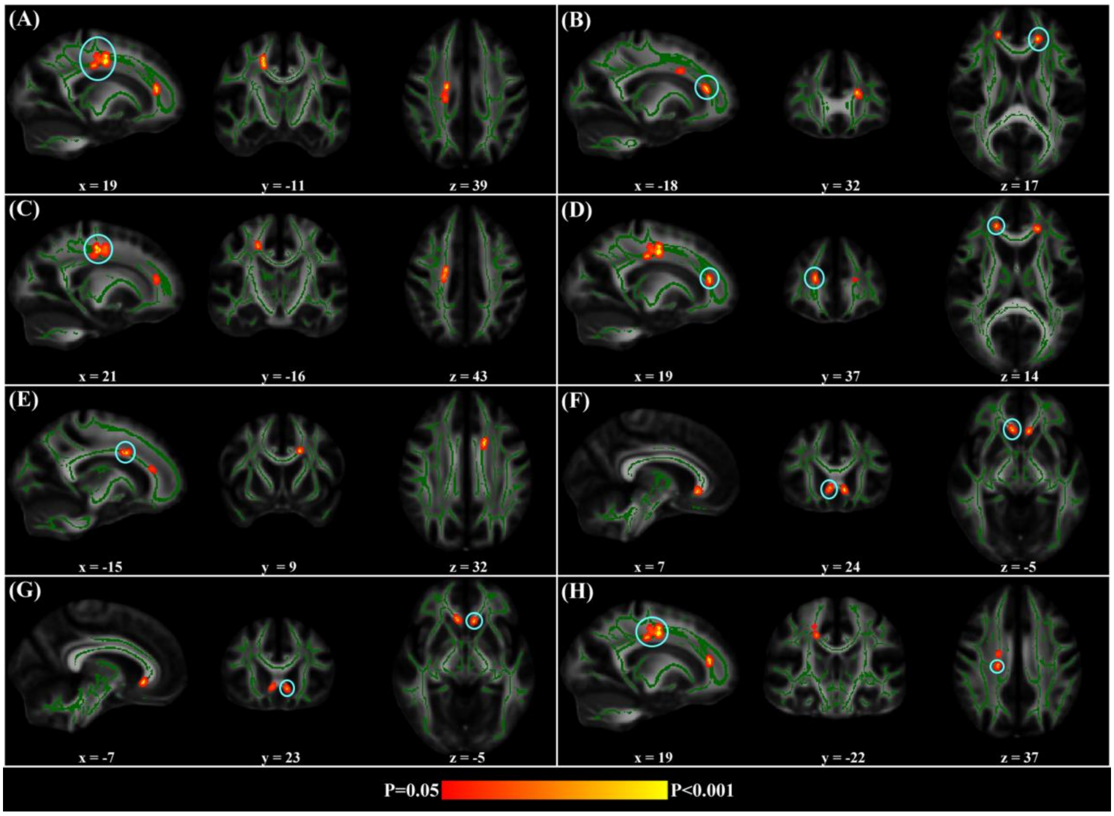

Fig. S4. Significant positive age-vs-RD correlations across subjects. *The threshold was $p < 0.05$ (FWE corrected), and only clusters not smaller than 10 mm^3 were shown. The correlation map was superimposed on the FA template in the MNI space (http://fsl.fmrib.ox.ac.uk/fsl/fslwiki/FMRIB58_FA). Green color indicates the FA skeleton mask, and red-yellow color indicates significant positive correlations. No significant negative correlation was observed. Significant positive age-vs-RD correlations were observed in the bilateral genu of the corpus callosum (B, D, F, G), the left body of the corpus callosum (E) and the right corticospinal tract (A, C, H), as were highlighted by circles when there were more than one cluster on the slice.*
